# Presence of p25alpha-Domain in Seed Plants (Spermatophyta): Microbial/Animal Contaminations and/or Orthologs

**DOI:** 10.3390/life13081664

**Published:** 2023-07-30

**Authors:** Ferenc Orosz

**Affiliations:** Institute of Enzymology, Research Centre for Natural Sciences, 1117 Budapest, Hungary; orosz.ferenc@ttk.hu

**Keywords:** apicomplexa, apicortin, genomic contamination, Spermatophyta, TPPP, early-branching fungi

## Abstract

Genome and transcriptome assembly data often contain DNA and RNA contaminations from external organisms, introduced during nucleotide extraction or sequencing. In this study, contamination of seed plant (Spermatophyta) transcriptomes/genomes with p25alpha domain encoding RNA/DNA was systematically investigated. This domain only occurs in organisms possessing a eukaryotic flagellum (cilium), which seed plants usually do not have. Nucleotide sequences available at the National Center for Biotechnology Information website, including transcriptome shotgun assemblies (TSAs), whole-genome shotgun contigs (WGSs), and expressed sequence tags (ESTs), were searched for sequences containing a p25alpha domain in Spermatophyta. Despite the lack of proteins containing the p25alpha domain, such fragments or complete mRNAs in some EST and TSA databases were found. A phylogenetic analysis showed that these were contaminations whose possible sources were microorganisms (flagellated fungi, protists) and arthropods/worms; however, there were cases where it cannot be excluded that the sequences found were genuine hits and not of external origin.

## 1. Introduction

Genome and transcriptome assembly data often contain DNA and RNA contaminations, originating from external organisms introduced during nucleotide extraction or sequencing. A large-scale search identified more than 2,000,000 contaminated entries in GenBank and other databases [1]. Consequently, database searches can lead to erroneous results, due to these impurities. It would be best to avoid this through careful sampling beforehand, but if this fails or is unavoidable, through subsequent bioinformatics filtering. Human-derived impurities and other laboratory contaminants such as *E. coli* and cloning vectors can be effectively eliminated using highly efficient computational filters applied to the draft sequences [2]. However, other contaminations are more difficult to identify, especially if no reference genome or transcriptome is available. For example, published mammalian and avian genomes and proteomes have been shown to be contaminated with genes/proteins of apicomplexan parasite origin [3]. Through the spread of next-generation sequencing, this has become a common problem, due to the vast amount of reads, which are generally short and of low quality in these projects [4].

In contrast to animals [5], relatively few such studies have been conducted on plants, but there are a few where insect or fungal contamination was identified [6,7,8]. Zhu et al. found a number of olfactory, odorant-binding, and chemosensory proteins in plant transcriptomes, due to insect contamination [6]. In another study, fungal contamination (from *Aureobasidium pullulans*) was found in the genome of the domesticated olive [7]. The most detailed investigation was carried out by Saffer and Mattin [8]. It was shown that a large proportion of plant transcriptomes were contaminated with RNAs encoding POU domain proteins, which had not been described in plants before. They also found that draft genomes of *Humulus lupulus* and *Cannabis sativa* contained complete rDNA sequences derived from *Tetranychus* species (spider mite) [8]. These publications are based on data available in public databases and subsequently draw attention to the presence of contaminated sequences. Subsequent detection of contamination could be avoided if the authors of the experimental work performed these curation processes themselves, rather than focusing only on routine procedures (e.g., filtering out human contamination). An excellent recent example of this is Martín-Blázquez and colleagues’ paper about the in silico cleaning of the transcriptome of the fern *Vandenboschia speciosa*, as they themselves noticed “high inter-specific contamination levels due to the difficulty of collecting clean tissue” [9].

**Figure 1:**
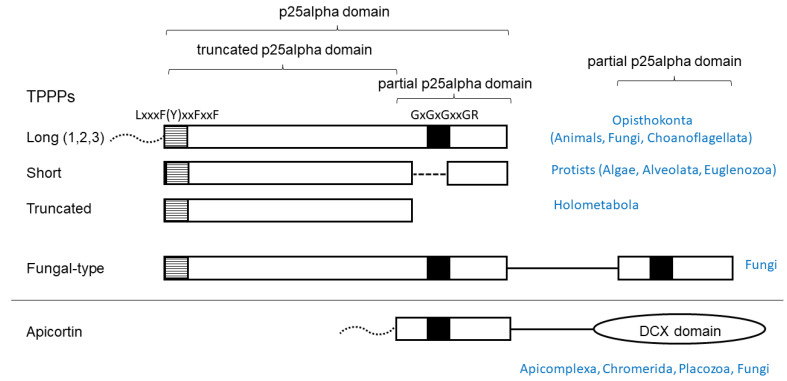
Schematic structure of TPPP-like proteins and their occurrence. Black and striped boxes indicate highly conservative sequence motifs. Dotted lines represent disordered regions of various length present in some species. (1,2,3) indicates that vertebrate genomes contain three paralogs.

In this study, contamination of plant transcriptomes with p25alpha domain encoding RNAs was systematically investigated. Whole-genome shotgun (WGS) contigs were also analyzed. Although EST (expressed sequenced tag) approaches have largely been superseded by whole genome and transcriptome sequencing, these were also searched for. The fact of contamination is relatively evident, or at least suspicious, if domains are found in a genome/transcriptome that is specific to other kingdoms of life. The p25alpha domain in TPPP-like proteins is one of these domains, and it is not known to occur at the protein level in land plants (Embryophyta) [10]. The reason for this is that this domain appears to be associated with the presence of flagellum/cilium, which is absent in most land plants [11]. The essential role of TPPP in the formation of flagella has been demonstrated in *Chlamydomonas reinhardtii*, a biflagellate green alga [12], and the apicomplexan parasite *Plasmodium yoelli* [13]. The most conserved part of the domain is the C-terminus, which contains a characteristic GXGXGXXGR sequence (Rossmann-like motif), making it relatively easy to recognize. Another characteristic sequence, L(F)xxxFxxF(Y)xxF, can be found at the very beginning of the domain (Figure 1).

TPPP proteins in which the full or partial p25alpha domain is present can be grouped according to the nature (completeness) of the domain [10] (Figure 1). The long (animal-type) TPPP is specific for Opisthokonta and is found in almost all animals, some flagellated fungi, and Choanoflagellate *Monosiga brevicollis* [10]. Some flagellated fungi contain fungal-type TPPPs (single copy or two paralogs) that have both a full domain and a partial domain (the C-terminal part), so that the Rossmann-like motif can be found twice in them [14]. The short TPPP is found in algae and protists (Alveolata, Euglenozoa), the C-terminal part of which is incomplete, while Rossmann-like motif is also absent [10]. In Endopterygota (Holometabola), insects undergoing metamorphosis, in addition to the long TPPP, there is a form in which the entire C-terminus is missing (“truncated” TPPP) [15]. Placozoan *Trichoplax adhaerens* (the only animal which lacks TPPP), Myzozoa (apicomplexans, chrompodellids, dinoflagellates, perkinsids), and some flagellated fungi contain apicortin in which the C-terminal portion (partial p25-alpha) is attached to a DCX domain [16,17].

Nucleotide sequences available on the National Center for Biotechnology Information (NCBI) website, including transcriptome shotgun assemblies (TSAs), WGS contigs, and ESTs, were searched for p25alpha-containing sequences in seed plants (Spermatophyta). The search was restricted to this clade, as there are no flagella or cilia in this phylogenetic unit, except for cycads and *Ginkgo biloba*, which possess flagellated male gametes. Despite the absence of proteins containing the p25alpha domain, such fragments or complete mRNAs were found in some EST and TSA databases. Possible sources of contamination were microorganisms (flagellated fungi, protists) and arthropods; however, there were cases where it cannot be excluded that the sequences found were genuine hits and not of external origin.

## 2. Materials and Methods

### 2.1. Database Homology Search

Accession numbers of protein and nucleotide sequences refer to the NCBI GenBank database, except if otherwise stated. The database search started with an NCBI Blast search [18] (http://www.ncbi.nlm.nih.gov/BLAST/, accessed on 20 March 2023). Sequences of various p25alpha-domain-containing proteins were used as queries against protein and nucleotide, including TSAs, WGSs, and ESTs databases, to find similar sequences in Spermatophyta using BLASTP and TBLASTN analyses, respectively. The queries were *Tetrahymena thermophila* XP_001023601, *Plasmodium falciparum* XP_001350760, *Babesia bovis* XP_001610770, *Trypanosoma brucei* XP_844424 for short TPPPs; *Drosophila melanogaster* NP_648881, *Caenorhabditis elegans* NP_491219, *Amphimedon queenslandica* XP_003384590, *M. brevicollis* XP_001743131 for long TTPPs; *Spizellomyces punctatus* XP_016604112, *Chytriomyces confervae* TPX65513, *Batrachochytrium dendrobatidis* XP_006680205, *Allomyces macrogynus* KNE68590 for fungal-type TPPPs; *D. melanogaster* NP_001097567, *Danaus plexippus* XP_032527880, *Nasonia vitripennis* XP_001604263 for truncated TPPPP; and *T. adhaerens* XP_002111209, *B. bovis* XP_001609847, *P. falciparum* XP_001351735, *Jimgerdemannia flammicorona* RUS30044.1, *S. punctatus* XP_016606225.1 for apicortins. The plant sequences found were used as queries in BLASTX search, to find the most similar sequence in the protein databases.

### 2.2. Phylogenetic Analysis

Bayesian analysis using MrBayes v3.1.2 [19] was performed to construct phylogenetic trees. Multiple alignments of sequences conducted using the Clustal Omega program [20] did not include the N-termini of the proteins, i.e., the amino acids before the p25alpha domain. Default priors and the WAG model [21] were used, assuming equal rates across sites. Two independent analyses were run with three heated and one cold chain (temperature parameter 0.2) for generations, as indicated in the Figure legends, with a sampling frequency of 0.01, and the first 25% of generations were discarded as burn-in. The two runs were convergent. A phylogenetic tree was drawn with the software Drawgram (http://evolution.genetics.washington.edu/phylip.html, accessed on 27 July 2015).

## 3. Results

### 3.1. Database Homology Search for the p25alpha-Domain in Streptophyta

Protein and nucleotide sequences available at the NCBI website, including TSAs, WGSs, and ESTs, were searched for p25alpha-containing sequences in seed plant (Spermatophyta) databases. Sequences of various proteins containing the p25alpha domain were used as queries (cf. Methods). No protein or WGS hits were found, but such fragments or complete mRNAs were found in some TSA and EST databases (Table 1). The initial BLAST search was performed with randomly selected proteins; therefore, the hits obtained may show low coverage and identity values. Thus, the sequences found in plants were used as queries in the BLASTX search, to find the most similar sequences in the protein databases. These hits are listed in Table 1. The results indicated that these sequences were of protist, fungal, or animal origin.

The hits were categorized by the type of the TPPP-like protein containing the p25alpha domain. Long, short, truncated, and fungal-type TPPPs and apicortins were found to be the best hits. In some cases, contamination was evident, where the sequence identity was 100% or close to this; for example, contamination of *Humulus lupulus* and *Myosoton aquaticum* originated from a spider mite (*Tetranychus urticae*) and an insect (*Frankliniella occidentalis)* long TPPP, respectively. The contamination of *Cenostigma pyramidale* came from an Endopterygota insect genus, *Anastrepha,* since the TSA GIYP01283228 was 98.29% identical to the truncated TPPP from *Anastrepha ludens*.

### 3.2. Search for Further Contaminations

Some species (*B. papyrifera*, *T. polonicum*, *O. sativa, N. tabacum*) had more than one p25alpha-domain-containing sequence (Table 1). This would be especially difficult to explain if one considered them as genuine sequences. The birch (*B. papyrifera*) transcriptome [22] contained one and two TSA sequences, corresponding to the short (GEIC01017558) and the fungal-type (GEIC01019177, GEIC01019178) TPPPs, respectively. This made it rational to check whether the *B. papyrifera* transcriptome contained any more potential contaminating sequences. As a test, the TSA sequences GEIC01017550–GEIC01017560 and GEIC01019170–GEIC01019180 (i.e., a window of ten sequences around the p25alpha hits) were used as queries to find the most similar proteins (Table 2). The best match in only two out of twenty cases was a plant sequence. Fungi gave the best results in twelve cases, Oomycota in three cases, and other species in another three cases. For five fungi and one Oomycota sequence, both the identity and the query cover were higher than 90%; in four cases, these were higher than 97%. Three out of the six represented the Ascomycota fungus, *Dactylonectria macrodidyma*. These values obviously reflect contaminations.

### 3.3. Phylogenetic Analysis

**Figure 2:**
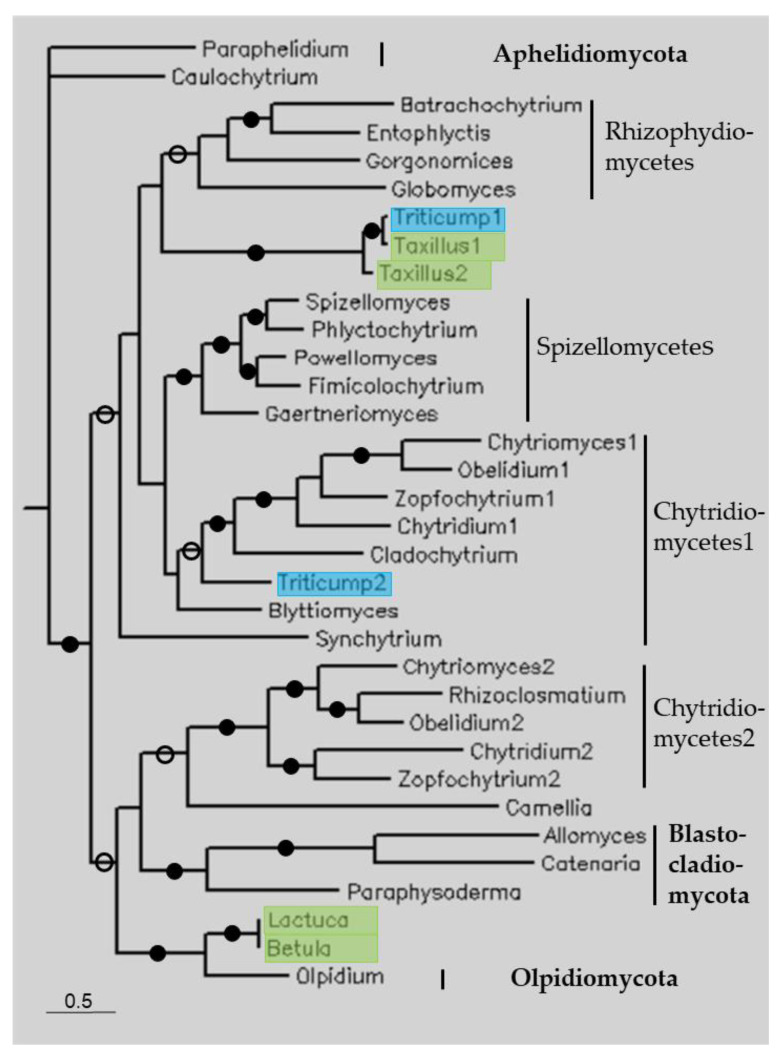
The phylogenetic tree of some fungal-type TPPPs constructed using Bayesian analysis [19]. The number of generations was 1.2 × 10^−6^. Full and open circles at a node indicate that the branch was supported by the maximal Bayesian posterior probability (BPP) and ≥0.95 BPP, respectively. All other branches were supported by a BPP ≥ 0.5. The accession numbers of proteins are listed in Table 1 and Table S1. Color code: blue, Magnoliopsida class, eudicotyledons; green, Magnoliopsida class, monocotyledons (Liliopsida).

**Figure 3:**
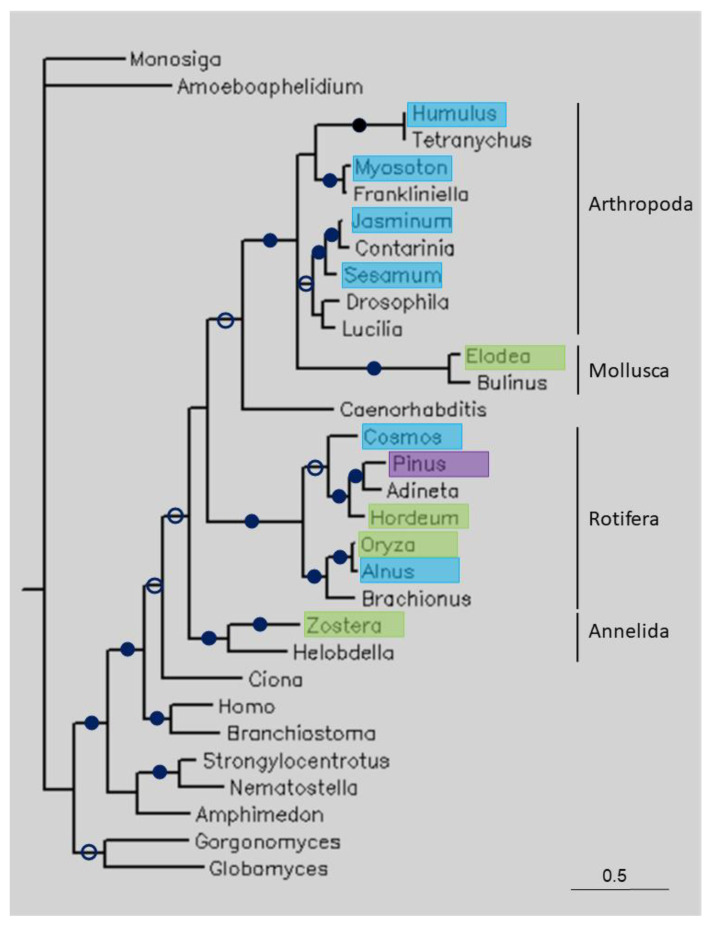
Phylogenetic tree of some long TPPPs constructed using Bayesian analysis [19]. The number of generations was 1.2 × 10^−6^. Full and open circles at a node indicate that the branch was supported by the maximal Bayesian posterior probability (BPP) and ≥0.95 BPP, respectively. All the other branches were supported by a BPP ≥0.5. The accession numbers of proteins are listed in Table 1 and Table S1. Color code: blue, Magnoliopsida class, eudicotyledons; green, Magnoliopsida class, monocotyledons (Liliopsida); pink, Pinopsida class.

**Figure 4:**
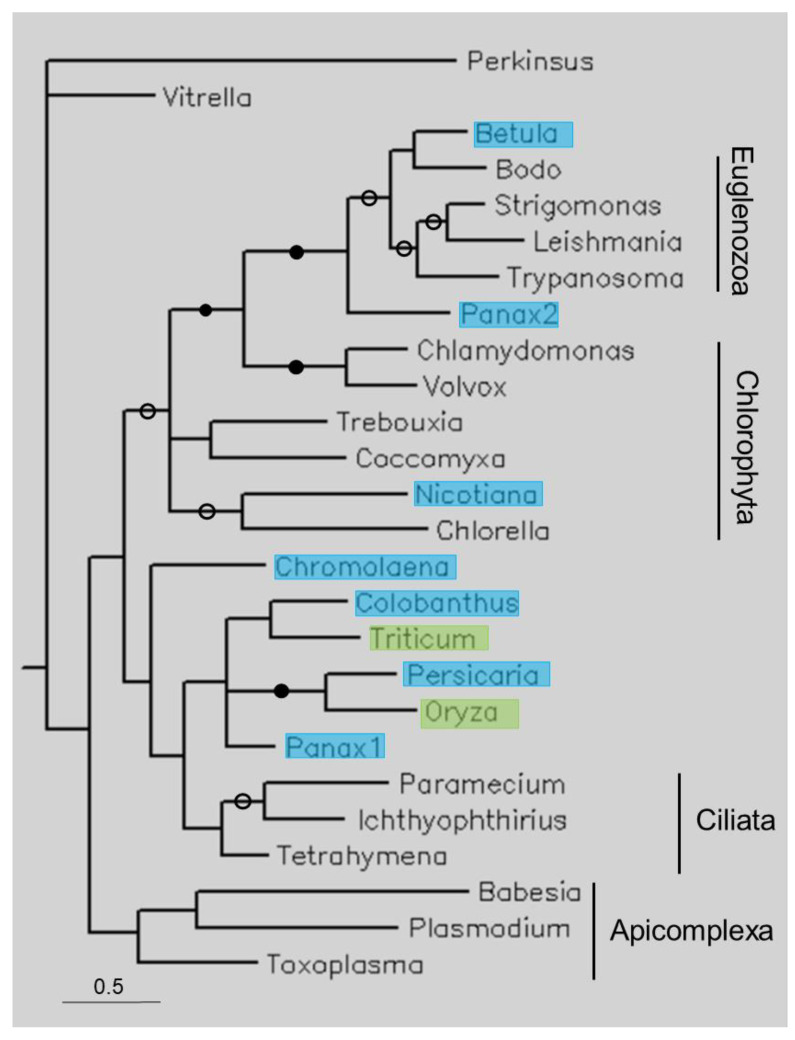
Phylogenetic tree of some short TPPPs constructed using Bayesian analysis [19]. The number of generations was 2.4 × 10^−6^. Full and open circles at a node indicate that the branch was supported by the maximal Bayesian posterior probability (BPP) and ≥0.95 BPP, respectively. All the other branches were supported by a BPP ≥ 0.5. The accession numbers of proteins are listed in Table 1 and Table S1. Color code: blue, Magnoliopsida class, eudicotyledons; green, Magnoliopsida class, monocotyledons (Liliopsida).

**Figure 5:**
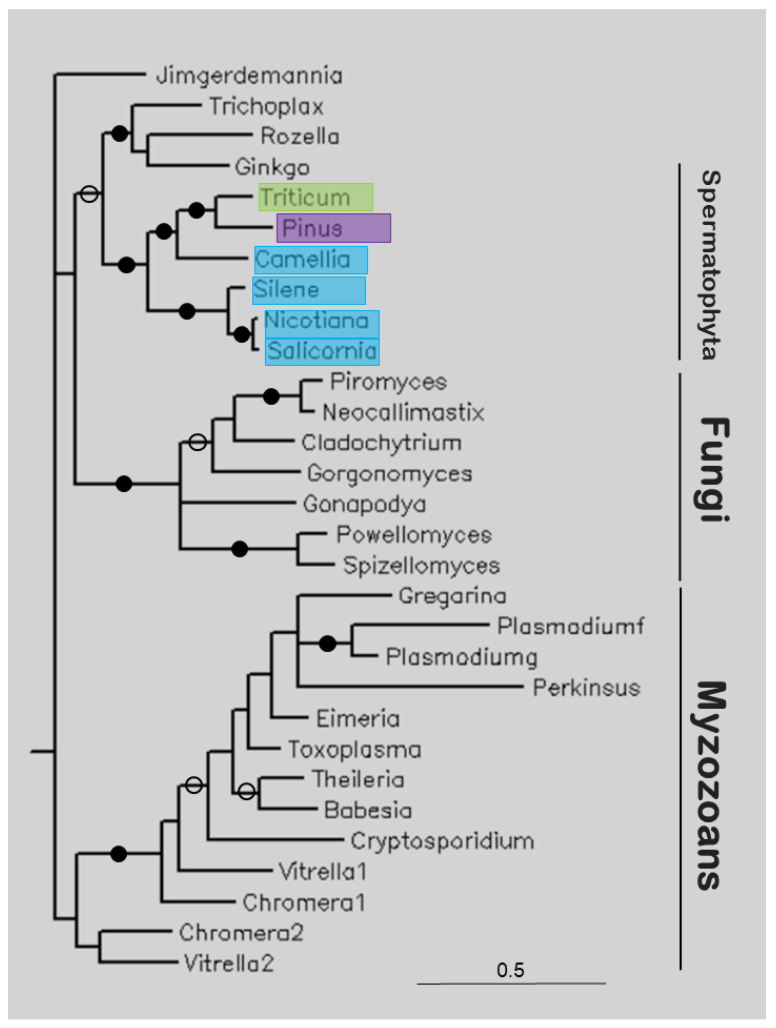
The phylogenetic tree of some apicortins constructed through Bayesian analysis [19]. The number of generations was 2.4 × 10^−6^. Full and open circles at a node indicate that the branch was supported by the maximal Bayesian posterior probability (BPP) and ≥0.95 BPP, respectively. All the other branches were supported by a BPP ≥ 0.5. The accession numbers of proteins are listed in Table 1 and Table S1. Color code: blue, Magnoliopsida class, eudicotyledons; green, Magnoliopsida class, monocotyledons (Liliopsida); pink, Pinopsida class.

Phylogenetic trees were constructed through Bayesian analysis using the sequences listed in Table 1, as well as those of some reference genomes (Figure 2, Figure 3, Figure 4 and Figure 5). Figure 2 shows a constructed tree of some fungal-type TPPPs. The tree follows the species phylogeny; the fungal phyla, Aphelidiomycota, Blastocladiomycota, Chytridiomycota, and Olpidiomycota form separate clades; within Chytridiomycota, the classes Chytridiomycetes, Rhizophydiomycetes, and Spizellomycetes are also separated. Species in Chytridiomycetes have two paralogous fungal-type TPPPs [14], thus forming two clades. The plant sequences are within the fungal clades. Although *Triticum polonicus* and *Taxillus chinensis* belong to different orders, they are sisters to each other and together are sisters to Rhizophydiomycetes. *Lactuca serriola* and *Betula papyrifera*, which have the same (!) sequence, are sisters to Olpidiomycota, while another *T. polonicus* sequence is within Chytridiomycetes.

Figure 3 shows a tree of some long TPPPs. The Choanoflagellata (*Monosiga brevicollis*), fungi (*Amoeboaphelidium protococcorum*, *Globomyces pollinis-pini*, *Gorgonomyces haynaldii*), and animal TPPPs formed separate clades. The plant sequences are found within animals, namely in groups representing the phyla Arthropoda, Mollusca, Rotifera and Annelida. Within Rotifera, plant sequences from the classes Magnoliopsida (both eudicotyledons and monocotyledons) and Pinopsida can be found. Eudicotyledon *Oryza sativa* and monocotyledon *Alnus glutinosa* are sisters.

Figure 4 shows the tree of a few of short TPPPs. The tree follows the species phylogeny; the phyla Apicomplexa, Chlorophyta, Ciliata, and Euglenozoa form separate clades. Plant sequences occupy different positions. *B. papyrifera* is within, *Panax ginseng* is sister to Euglenozoa, and *Nicotiana tabacum* is within Chlorophyta. Several other plant sequences are sisters to Ciliata TPPPs. Within this clade the eudicotyledons and monocotyledons are not separated.

Figure 5 shows the tree of several apicortins. Plant (Spermatophyta) sequences are sister to a clade containing apicortins of *T. adhaerens, Rosella allomycis,* and *G. biloba* (itself a Spermatophyta), and together they are sister to Fungi, and these clades together are sister to Myzozoan apicortins. This latter clade includes apicomplexan, chromerid, and perkinsoan proteins.

## 4. Discussion

In this study, the sequences of seed plants (Spermatophyta) deposited in the NCBI databases were systematically examined for the presence of the p25alpha domain. This domain is found in TPPP-like proteins, which are absent in land plants [10,23]. The reason for this is that the p25alpha domain is connected to the presence of flagellum/cilium, which was lost from most land plants during evolution [11]. The search was restricted to the Spermatophyta, as only two classes, Ginkgoopsida and Cycadopsida, contain species with flagellum/cilium; thus, except for these, the occurrence of TPPP-like genes/proteins is not expected. Although no such proteins were found, fragments or complete mRNAs were found in some TSA and EST databases (Table 1).

These nucleotide sequences showed homology, and in a few cases identity, with long, short, truncated, and fungal-type TPPPs or apicortins. In the case of sequence identity, contamination was evident, and its source was obvious (*H. lupulus* and *M. aquaticum* transcriptomes were contaminated with *T. urticae* and *F. occidentalis* sequences, respectively). In both cases, the contamination was long TPPP. In the only case where truncated TPPP was found in a plant transcriptome, *C. pyramidale,* the situation was very similar; the sequences were almost identical, with only two conservative substitutions in the translated RNA sequence. The source is given by the best hit in Table 1 (*A. ludens*) or is from the same genus, *Anastrepha.*

In the above-mentioned cases, as well as in the case of the next highest identity value (92%, *Jasminum sambac* and *Contarinia nasturtii*), the best hit was a long TPPP homologue sequence and the potential contaminator was an Arthropoda, mostly an insect. One of the few previous papers that looked at the contamination of plant transcriptomes found some of these plant–arthropod pairings. *H. lupulus* contained complete rDNA sequences originating from *T. urticae* [8]. A *J. sambac* TSA (GHOY01040882) was identical at 98% to a *C. nasturtii* mRNA (XM_031763638) [8]. Another study found *H. lupulus* as the plant that was the most contaminated by insect chemosensory proteins, while *F. occidentalis* was identified as one of the sources [6]. The presence of arthropod-derived contaminations in plants is therefore not uncommon. These arthropod species are often pests of various plants and secretions left behind from saliva may cause the contamination [6,8].

In general, in the case of the long TPPPs (and the only truncated one), the plant sequences had significant similarities and coverage values as the animal sequences. The identity values were much higher than those for fungal and short TPPPs or apicortins and generally higher than 75% (Table 1) (the only exception was *Zostera noltei* vs. *Helobdella robusta*.) In addition to arthropods, worms (Rotifera, Annelida) and molluscs were also among the sources of contamination. The high, but less than 100%, values indicate that the contamination was probably related to other, close species whose transcriptome (genome) is not or not completely available in the databases. Out of twenty randomly selected *B. papyrifera* TSA sequences, six certainly appeared to be fungal or Oomycota contamination, but the number was probably higher (Table 2). We cannot generalize based on this, as this would require a systematic analysis of the transcriptome; however, the high rate of contamination highlights the importance of the issue.

Where the identity and coverage of the sequences is not as high as in the above-mentioned cases, the explanation for the presence of unexpected sequences in genomes/transcriptomes is not as straightforward. These similarities may be due to different factors: they can be true orthologous sequences (conservation) or they may be the consequence of horizontal (lateral) gene transfer (HGT) or contamination of sequencing data. Phylogenetic analysis can help to distinguish between these possibilities. If the homology between plant and other, e.g., animal or fungal, sequences was due to orthology, we would expect plant species to be located outside of animals or fungi in the tree. If the plant sequence is located within another clade, then contamination or HGT may have occurred. HGT does not often happen between higher eukaryotes, such as between distantly related organisms such as arthropods and plants, although it is difficult to rule it out completely. Recently, these kind of reports of HGT have been accumulating, but in the opposite direction, from plants to arthropods [24]. However, a high sequence identity usually suggests contamination, as HGT would have occurred some time ago and the sequence may have changed significantly since then. For the long TPPPs, the plant sequences were located within various animal clusters (Figure 3), confirming that contamination occurred.

The other TPPP-like proteins did not show such a high similarity to the plant sequences, although the identity mostly exceeded 40%. Fungal-type TPPPs are specific to fungi. The phylogenetic tree of fungal-type TPPPs shows that plant sequences were located within clades of various fungal phyla, thus a real orthology can be ruled out (Figure 2). The position of the plant sequences supports that sequence contamination occurred. *T. polonicus* is an eudicotyledon and *T. chinensis* is a monocotyledon, they are sisters to each other and together are sisters to Rhizophydiomycetes. Similarly, *L. serriola* and *B. papyrifera* are sisters to each other and together are sisters to Olpidiomycota. In fact, the latter two plant sequences are identical. This is unlikely in the case of HGT, but it can easily be understood if the source of the contamination is the same. However, the source of the contaminations cannot be identified, since the sequence identities are far below 100%.

Unlike in previous cases, the analysis of short TPPPs was more complex, as they are not specific to one or two phyla but occur in many. The plant sequences do not form a separate clade but occupy different positions on the tree (Figure 4). *B. papyrifera* is within Euglenozoa, *P. ginseng* is sister to Euglenozoa, and *N. tabacum* is within Chlorophyta. There is no plant sequence within Apicomplexa. Several other plant sequences are sisters to Ciliata TPPPs, although the BPP support is not high. Within this clade the eudicotyledons and monocotyledons are not separated. The positions of these plant sequences do not support that they are true orthologs. The identities are relatively high, 60–70%; they are only higher for long and truncated TPPPs, where the contaminations were of animal origin. Most plant nucleotide sequences correspond to whole proteins (*B. papyrifera, Colobanthus quitensis, P. ginseng* 1, *N. tabacum*, *O. sativa*, and *Triticum aestivum*) and show sequence elements very characteristic of short TPPPs. However, it is questionable how contamination or HGT could have happened. Unlike other TPPP-like proteins, the phylogenetic occurrence of short TPPPs has not been systematically investigated, except for myzozoan species [17]. However, a rough examination has shown that they are common in some algae, ciliates, and euglenozoan [10]. Thus, short TPPPs only occur in various microorganisms that are not in connection with plants; for example, *B. papyrifera* is sister to *Bodo saltans*, a Euglenozoa. 

An explanation may arise: previously, it was found that stramenopiles, more precisely Oomycota, do not have short TPPP but contain *multidomain* proteins that have a short p25alpha domain [10]. Oomycetes are pathogenic parasites of plants; thus, they have the potential to contaminate plant genomes/transcriptomes (cf. also Table 2). However, a BLAST search indicated that the potential short p25alpha domain contaminants found in the present work (Table 1) are unlikely to be of Oomycota origin. The best Oomycota hits in terms of coverage and percentage identity gave lower values than those belonging to other phylogenetic units (Table S2). All in all, I must leave this question open.

The study of apicortins seems to represent another scenario. This protein has been found in the placozoan animal *T. adhaerens* [25], in flagellated fungi [14], and in myzozoans (Apicomplexa [25], chromerids [16], Perkinsozoa [26], and dinoflagellates [17]). In the present study, it was found at nucleotide level, as TSA or EST. The majority of the hits contained the full sequence of apicortin (*Camellia sinensis, G. biloba*, *N. tabacum*, *Triticum polonicum*). Of these species, only ginkgo (*G. biloba*) has cilia; its spermatozoa are moved by thousands of cilia [27]. Our phylogenetic analysis showed that the Spermatophyta sequences form a separate clade that is sister to Fungi, and these clades together are sisters to myzozoan apicortins (Figure 5) (*G. biloba* sequence, with two other apicortins, is a sister position to the other Spermatophyta sequences). Within the Spermatophyta clade, eudicotyledons are separated from monocotyledons (Liliopsida). The identity values of plant sequences compared to other apicortins are about 50%. There are apicortins of animal, fungal, and chromerid origin among the most similar hits. Since the plant species are outside of animals or fungi in the tree, it can be assumed that these sequences are not the results of contamination or HGT but represent genuine apicortins that occur as a kind of relic in this non-flagellated species. A similar phenomenon occurs in non-flagellated Mucoromycota fungi [14].

## 5. Conclusions

Detection of contaminants from organisms without a fully sequenced genome is a challenge. In the case of plants, this topic seems to be quite neglected. However, the investigation of (draft) genomes and transcriptomes for potential contamination has several advantages. (i) It can filter out true contamination that would lead to erroneous conclusions about the functions of the organism. (ii) It may lead to the discovery of new species for which there are examples [3] and suggestions for such a use [28,29,30]. (iii) It can lead to the identification of parasites and plant pests of the given species. (iv) If a “guest sequence” not specific to a given species or phylogenetic unit turns out to be a true match, it may be suitable for drawing important evolutionary conclusions, either as a result of HGT or as an evolutionary consequence.

In this study, possible contaminations of Spermatophyta genomes/transcriptomes/proteomes with sequences containing the p25alpha domain were investigated. This domain occurs almost exclusively in species with eukaryotic flagellum (cilium), which seed plants usually do not have. The domain was found at the nucleotide level as TSA or EST. For the different proteins containing the p25alpha domain, different results were obtained as the reason for the presence of the domain. The occurrence of sequences corresponding to long and truncated TPPPs can be attributed to animal contaminants, whereas fungal-type TPPP contaminating sequences are derived from fungi. For the short TPPPs, which are only found in microorganisms (Apicomplexa, Ciliata, Clorophyta, Euglenoza), no clear answer could be given as the cause of the presence of this domain. Apicortins are probably true hits and might be orthologs of this protein. The latter is quite surprising and further studies are needed to find out what their function might be.

## Figures and Tables

**Table 1:** Nucleotide sequences containing the p25alpha domain in seed plants. The best protein hits of each of these nucleotides in other organisms are also given.

Plant Species	Order	Accession No ^1^	Species	Accession No	Cover %	Identity %
				Fungal-type		
*Lactuca serriola*	Asterales ^2^	JO041594	*Spizellomyces punctatus* ^3^	XP_016604112	92	42.81
*Taxillus chinensis* ^1^	Santalales	GHNL01117630	*S. punctatus*	XP_016604112	61	40.51
*T. chinensis* ^2^	Santalales	GHNL01117629	*S. punctatus*	XP_016604112	89	38.01
*Betula papyrifera*	Fagales	GEIC01019178 ^4^ GEIC01019177 ^4^	*Quaeritorhiza haematococci*	KAJ3085108	76 82	49.74
*Triticum polonicum* ^1^	Poales	GEDP01099476	*S. punctatus*	XP_016604112	60	40.51
*T. polonicum* ^2^	Poales	GEDP01150747	*Powellomyces hirtus*	TPX57673	85	61.73
				Long		
*Humulus lupulus*	Rosales	GAAW01037957	*Tetranychus urticae*	XP_015786377	65	100
*Myosoton aquaticum*	Caryophyllales	GGTY01056430	*Frankliniella occidentalis*	XP_026285276	60	100
*Jasminum sambac*	Lamiales	GHOY01138054	*Contarinia nasturtii*	XP_031639744	51	92.02
*Cosmos caudatus*	Asterales	GJBF01051822	*Adineta steineri*	CAF1404786	91	79.43
*Zostera noltei*	Alismatales	HACV01012836	*Helobdella robusta*	XP_009008741	46	51.23
*Elodea nuttallii*	Alismatales	GBEN01147374	*Bulinus truncatus*	KAH9498372	71	88.24
*Pinus lambertiana*	Pinales	GEUZ01024616	*Adineta vaga*	UJR13967	99	82.22
*Oryza sativa*	Poales	CT849204 *	*Brachionus calyciflorus*	CAF0835781	99	76.09
*Hordeum vulgare*	Poales	BM815954 *	*Adineta vaga*	UJR13967	100	80.00
*Alnus glutinosa*	Fagales	FQ350563 *	*B. calyciflorus*	CAF0835781	100	75.62
*Sesamum indicum*	Lamiales	JK067166 *^,5^ JK062224 *^,5^	*Lucilia cuprina*	XP_023301338	100	84.75
				Short		
*Panax ginseng* ^1^	Apiales	GDQW01019137	*Tetrahymena thermophila*	XP_001023601 ^7^	98	70.27
*P ginseng* ^2^	Apiales	GDQW01005616	*Trypanosoma brucei*	XP_011772860	95	62.50
*B. papyrifera*	Fagales	GEIC01017558	*Bodo saltans*	CUE71550	90	73.57
*Nicotiana. tabacum*	Solanales	AM817762 *^,6^ AM824543 *^,6^	*Coccomyxa* sp.	BDA43246	100	47.24
*Colobanthus quitensis*	Caryophyllales	GCIB01125581	*T. thermophila*	XP_001023601	98	68.03
*Persicaria minor*	Caryophyllales	GALN01112310	*T. thermopila*	XP_001023599	79	58.62
*Chromolaena odorata*	Asterales	GACH01135300	*Paramecium sonneborni*	CAD8055868	100	60.34
*O. sativa*	Poales	CT850609 *	*T. thermophila*	XP_001023601	92	58.11
*Triticum aestivum*	Poales	CD868723 *	*T. thermophila*	XP_001023599	96	61.22
				Truncated		
*Cenostigma pyramidale*	Fabales	GIYP01283228	*Anastrepha ludens*	XP_053956472	100	98.29
				Apicortin		
*Camellia sinensis*	Ericales	GFMV01019718	*Vitrella brassicaformis*	CEM06711	44	41.83
*N. tabacum*	Solanales	AM844195 *	*Jimgerdemannia flammicorona*	RUS30044	73	50.67
*Silene dioica*	Caryophyllales	GFCH01066796	*J. flammicorona*	RUS30044	100	48.57
*Salicornia europaea*	Caryophyllales	GAMH01042109	*Rosella allomycis*	EPZ32946	85	51.00
*T. polonicum*	Poales	GEDP01156285	*Trichoplax adhaerens*	XP_002111209	65	47.24
*Ginkgo biloba*	Ginkgoales	GHLL01465948	*T. adhaerens*	XP_002111209	78	54.49
*Pinus flexilis*	Pinales	GHWB01415589	*J. flammicorona*	RUS30044	91	48.78

^1^ Accession numbers in the third column refer to TSAs or ESTs *. ^2^ Color code: blue, Magnoliopsida class, eudicotyledons; green, Magnoliopsida class, monocotyledons (Liliopsida); pink, Pinopsida class; no color: Ginkgoopsida class. ^3^ Color code: yellow, fungi; blue, animals; red, ciliates; green, Euglenozoa; gray, Chlorophyta; no color: chromerids. ^4^ Practically the same sequences. ^5^ Practically the same sequences. ^6^ Practically the same sequences. ^7^ This sequence was used for Figure 4.

**Table 2:** Best hits for several *Betula papyrifera* TSAs found through a BLASTX search in the NCBI protein database.

Accession Number	Best Hit				
	Accession Number	Species	Phylum	Query Cover, %	Identity, %
GEIC01019180	KAH7131324	*Dactylonectria macrodidyma*	Ascomycota	72	86.84
	KFY33973	*Pseudogymnoascus* sp.	Ascomycota	73	61.84
GEIC01019179	XP_015895121	*Ziziphus jujuba*	Streptophyta	95	78.67
GEIC01019178	KAJ3085108	*Quaeritorhiza haematococci*	Chytridiomycota	76	49.74
	KAJ3185965	*Gaertneriomyces* sp.	Chytridiomycota	99	42.13
GEIC01019177	KAJ3085108	*Quaeritorhiza haematococci*	Chytridiomycota	82	49.74
	KAJ3031848	*Rhizophlyctis rosea*	Chytridiomycota	91	42.17
GEIC01019176	XP_018131936	*Pseudogymnoascus verrucosus*	Ascomycota	**99**	**100**
GEIC01019175	KAF1315998	*Globisporangium splendens*	Oomycota	**99**	**90.43**
GEIC01019174	KIJ40333	*Sphaerobolus stellatus*	Basidiomycota	65	39.13
GEIC01019173	PNP46136	*Trichoderma gamsii*	Ascomycota	**99**	**97.53**
GEIC01019172	KAH7141718	*Dactylonectria macrodidyma*	Ascomycota	**98**	**99.34**
GEIC01019171	KAH7121618	*Dactylonectria macrodidyma*	Ascomycota	**98**	**94.90**
GEIC01019170	TFK80833	*Polyporus arcularius*	Basidiomycota	40	61.83
	XP_008038329	*Trametes versicolor*	Basidiomycota	47	54.25
GEIC01017560	KAE8022277	*Carpinus fangiana*	Streptophyta	**100**	**91.74**
GEIC01017559	CUE71550	*Bodo saltans*	Euglenozoa	86	75.56
	EPY25997	*Angomonas deanei*	Euglenozoa	94	63.27
GEIC01017558	CUE71550	*Bodo saltans*	Euglenozoa	90	73.57
GEIC01017557	ELT89137	*Capitella teleta*	Annelida	87	53.04
GEIC01017556	KAH7144037	*Dactylonectria macrodidyma*	Ascomycota	**99**	**98.57**
GEIC01017555	XP_022516040	*Fonsecaea monophora*	Ascomycota	22	100
GEIC01017554	XP_022516040	*Fonsecaea monophora*	Ascomycota	27	100
GEIC01017553	-	*-*	-	-	-
GEIC01017552	-	*-*	-	-	-
GEIC01017551	TYZ60970	*Pythium brassicae*	Oomycota	99	74.36
GEIC01017550	TYZ60970	*Pythium brassicae*	Oomycota	81	75.76
	KAG7389301	*Phytophthora pseudosyringae*	Oomycota	83	62.69

Color code: yellow—fungi, blue—animals, deep blue—stramenopiles, green—Euglenozoa, no color—plants. Bold numbers indicate that both the identity and the query cover were higher than 90%.

## Data Availability

All data are available in the paper and in the supplementary material.

## Supplementary Materials

The following supporting information can be downloaded at https://www.mdpi.com/article/10.3390/life13081664/s1, Table S1: Accession numbers of proteins shown in Figure 2, Figure 3, Figure 4 and Figure 5. Table S2: Nucleotide sequences containing short p25alpha domain in seed plants (Cf. Table 1). The best protein hits of each of these nucleotides in Ooymycota are given.
