# Supplementary material for: Presence of p25alpha-Domain in Seed Plants (Spermatophyta): Microbial/Animal Contaminations and/or Orthologs
Source: Life (Basel). 2023 Jul 30;13(8):1664. doi: 10.3390/life13081664 (PMC10455874; doi:10.3390/life13081664)
Supplement: Supplementary file 1 [file life-13-01664-s001.zip › life-2493447-supplementary.pdf]

Table S1. Accession Numbers of proteins shown in Figures 2-5[1]

Name on the Figures	Species	Phylum	NCBI Accession No.
**Figure 2**			
Allomyces	*Allomyces macrogynus*	Blastocladiomycota	KNE68590
Amoeboaphelidium	*Amoeboaphelidium protococcarum*	Aphelidiomycota	KAI3631655
Batrachochytrium	*Batrachochytrium dendrobatidis*	Chytridiomycota	XP_006680205
Blyttiomyces	*Blyttiomyces helicus*	Chytridiomycota	RKO89614
Catenaria	*Catenaria anguillulae*	Blastocladiomycota	ORZ33943
Chytridium1	*Chytridium lagenaria*	Chytridiomycota	KAI8852913
Chytridium2	*Chytridium lagenaria*	Chytridiomycota	KAI8846486
Chytriomyces1	*Chytriomyces confervae*	Chytridiomycota	TPX65886
Chytriomyces2	*Chytriomyces confervae*	Chytridiomycota	TPX72533
Cladochytrium	*Cladochytrium replicatum*	Chytridiomycota	KAI8806435
Entophlyctis	*Entophlyctis helioformis*	Chytridiomycota	KAI8924042
Fimicolochytrium	*Fimicolochytrium jonesii*	Chytridiomycota	XP_052926454
Gaertneriomyces	*Gaertneriomyces semiglobifer*	Chytridiomycota	KAI9004733
Globomyces	*Globomyces pollinis-pini*	Chytridiomycota	KAI8893030
Gorgonomyces	*Gorgonomyces haynaldii*	Chytridiomycota	KAI8912588
Obelidium1	*Obelidium mucronatum*	Chytridiomycota	KAI9351240
Obelidium2	*Obelidium mucronatum*	Chytridiomycota	KAI9342285
Olpidium	*Olpidium bornovanus*	Olpidiomycota	KAG5460860+ KAG5458366
Paraphelidium	*Paraphelidium tribonemae*	Aphelidiomycota	TRINITY_DN24782[2]
Paraphysoderma	*Paraphysoderma sedebokerense*	Blastocladiomycota	KAI9140125
Phlyctochytrium	*Phlyctochytrium arcticum*	Chytridiomycota	KAI9095929
Rhizoclosmatium	*Rhizoclosmatium globosum*	Chytridiomycota	ORY45507
Synchytrium	*Synchytrium microbalum*	Chytridiomycota	XP_031024160
Zopfochytrium1	*Zopfochytrium polystomum*	Chytridiomycota	KAI9357369
Zopfochytrium2	*Zopfochytrium polystomum*	Chytridiomycota	KAI9325442
**Figure 3**			
Amoeboaphelidium	*Amoeboaphelidium protococcorum*	Aphelidiomycota	KAI3631655
Amphimedon	*Amphimedon queenslandica*	Porifera	XP_003384590
Branchiostoma	*Branchiostoma floridae*	Chordata	XP_002215000
Caenorhabditis	*Caenorhabditis elegans*	Nematode	NP_491219
Ciona	*Ciona intestinalis*	Chordata	XP_002124388
Drosophila	*Drosophila melanogaster*	Arthropoda	NP_648881
Helobdella	*Helobdella robusta*	Annelida	XP_009008741
Homo	*Homo sapiens*	Chordata	NP_776245
Globomyces	*Globomyces pollinis-pini*	Chytridiomycota	KAI8895260
Gorgonomyces	*Gorgonomyces haynaldii*	Chytridiomycota	KAI8912823
Monosiga	*Monosiga brevicollis*	Choanoflagellata	XP_001743131
Nematostella	*Nematostella vectensis*	Cnidaria	XP_001628751
Strongylocentrotus	*Strongylocentrotus purpuratus*	Echinodermata	XP_782492
**Figure 4**			
Babesia	*Babesia bovis*	Apicomplexa	XP_001610770
Chlamydonomas	*Chlamydomonas reinhardtii*	Chlorophyta	XP_001695016
Chlorella	*Chlorella variabilis*	Chlorophyta	XP_005848932
Ichthyophthirius	*Ichthyophthirius multifiliis*	Ciliata	XP_004035172

Leishmania	*Leishmania major*	Euglenozoa	XP_001686248
Perkinsus	*Perkinsus marinus*	Perkinsozoa	XP_002773110
Plasmodium	*Plasmodium falciparum*	Apicomplexa	XP_001350760
Strigomonas	*Strigomonas culicis*	Euglenozoa	EPY21771
Toxoplasma	*Toxoplasma gondii*	Apicomplexa	XP_002369913.
Trebouxia	*Trebouxia* sp. A1-2	Chlorophyta	KAA6422911
Vitrella	*Vitrella brassicaformis*	Chromerida	CEM02660
Volvox	*Volvox carteri*	Chlorophyta	XP_002946668
**Figure 5**			
Babesia	*Babesia bovis*	Apicomplexa	XP_001609847
Chromera1	*Chromera velia*	Chromerida	HBKZ010163093[3]
Chromera2	*Chromera velia*	Chromerida	JO8100183[3]
Cladochytrium	*Cladochytrium replicatum*	Chytridiomycota	KAI8808662
Cryptosporidium	*Cryptosporidium parvum*	Apicomplexa	XP_001388280
Eimeria	*Eimeria tenella*	Apicomplexa	XP_013231224
Gonapodya	*Gonapodya prolifera*	Monoblepharomycota	KXS19308
Gorgonomyces	*Gorgonomyces haynaldii*	Chytridiomycota	KAI8914275
Gregarina	*Gregarina niphandrodes*	Apicomplexa	XP_011128898
Neocallimastix	*Neocallimastix californiae*	Neocallimastigomycota	ORY36261
Perkinsus	*Perkinsus olseni*	Perkinsozoa	KAF4750811
Piromyces	*Piromyces finnis*	Neocallimastigomycota	ORX59328
Plasmodiumf	*Plasmodium falciparum*	Apicomplexa	XP_002808695
Plasmodiumg	*Plasmodium gallinaceum*	Apicomplexa	XP_028530755
Powellomyces	*Powellomyces hirtus*	Chytridiomycota	TPX59886
Spizellomyces	*Spyzellomyces punctatus*	Chytridiomycota	XP_016606225
Theileria	*Theileria annulat*	Apicomplexa	XP_952938
Toxoplasma	*Toxoplasma gondii*	Apicomplexa	XP_002364910
Vitrella1	*Vitrella brassicaformis*	Chromerida	CEM12737

[1]Accession Numbers listed in Table 1 are not shown here.

[2]Taken from https://figshare.com/articles/dataset/Commun_Biol_aphelid_datasets/7339469/1.

[3]TSA

Table S2. Nucleotide sequences containing short p25alpha domain in seed plants (Cf. Table 1). The best protein hits of each of these nucleotides in Ooymycota are given.

Plant species	Order	Accession Number[1]	Species	Accession Number	Query cover, %	Identity, %	(Query cover, %)[3]	(Identity, %)[3]
Panax ginseng 1	Apiales[2]	GDQW01019137	*Albugo laibachii*<br>*Aphanomyces astaci*	CCA17632<br>XP_009825419	31<br>51	47.37<br>33.93	(98)	70.27
Panax ginseng 2	Apiales	GDQW01005616	No hit in Oomycota				(95)	(62.50)
Betula papyrifera	Fagales	GEIC01017558	*Pythium oligandrum*<br>*Aphanomyces euteiches*	TMW64959<br>KAF0741241	43<br>65	41.10<br>31.62	(90)	(73.57)
Nicotiana. tabacum	Solanales	AM817762*	*Saprolegnia parasitica*<br>*Thraustotheca clavata*	XP_012194823<br>OQR96327	59<br>18	25.89<br>51.43	(100)	(47.24)
Nicotiana. tabacum	Solanales	AM824543*	*Saprolegnia parasitica*<br>*Thraustotheca clavata*	XP_012194823<br>OQR96327	66<br>20	25.89<br>51.43	(100)	(47.24)
Colobanthus quitensis	Caryophyllales	GCIB01125581	*Thraustotheca clavata*<br>*Pythium oligandrum*	OQR96327<br>TMW64959	41<br>45	43.75<br>37.14	(98)	(68.03)
Persicaria minor	Caryophyllales	GALN01112310	No hit in Oomycota				(79)	(58.62)
Chromolaena odorata	Asterales	GACH01135300	*Peronosclerospora sorghi*<br>*Lagenidium giganteum*	KAI9911451<br>DBA04893	64<br>86	50.00<br>42.50	(100)	(60.34)
Oryza sativa	Poales	CT850609*	No hit in Oomycota				(92)	(58.11)
Triticum aestivum	Poales	CD868723*	No hit in Oomycota				(96)	(61.22)

The best hits were searched by BLASTX program [18] in NCBI protein database using the nucleotids listed in the third column as queries. Search in NCBI nucleotide databases by BLASTN program [18] did not result in better hits. [1]Accession Numbers in the third column refer to TSAs or ESTs*. [2]Color code: blue, Magnoliopsida class, eudicotyledons; green, Magnoliopsida class, monocotyledons (Liliopsida). [3]Numbers in parenthesis are the values of the best hits in Table 1 (i.e., the absolute best hits, not only in Oomycota).
